# *Burkholderia phytofirmans* Inoculation-Induced Changes on the Shoot Cell Anatomy and Iron Accumulation Reveal Novel Components of *Arabidopsis*-Endophyte Interaction that Can Benefit Downstream Biomass Deconstruction

**DOI:** 10.3389/fpls.2016.00024

**Published:** 2016-01-29

**Authors:** Shuai Zhao, Hui Wei, Chien-Yuan Lin, Yining Zeng, Melvin P. Tucker, Michael E. Himmel, Shi-You Ding

**Affiliations:** ^1^Bioscience Center, National Renewable Energy LaboratoryGolden, CO, USA; ^2^National Bioenergy Center, National Renewable Energy LaboratoryGolden, CO, USA

**Keywords:** plant growth promoting bacteria (PGPB), *Burkholderia phytofirmans* PsJN, iron accumulation, essential mineral nutrients, biomass deconstruction, biomass conversion

## Abstract

It is known that plant growth promoting bacteria (PGPB) elicit positive effects on plant growth and biomass yield. However, the actual mechanism behind the plant-PGPB interaction is poorly understood, and the literature is scarce regarding the thermochemical pretreatability and enzymatic degradability of biomass derived from PGPB-inoculated plants. Most recent transcriptional analyses of PGPB strain *Burkholderia phytofirmans* PsJN inoculating potato in literature and *Arabidopsis* in our present study have revealed the expression of genes for ferritin and the biosynthesis and transport of siderophores (i.e., the molecules with high affinity for iron), respectively. The expression of such genes in the shoots of PsJN-inoculated plants prompted us to propose that PsJN-inoculation can improve the host plant's iron uptake and accumulation, which facilitates the downstream plant biomass pretreatment and conversion to simple sugars. In this study, we employed *B. phytofirmans* PsJN to inoculate the *Arabidopsis thaliana* plants, and conducted the first investigation for its effects on the biomass yield, the anatomical organization of stems, the iron accumulation, and the pretreatment and enzymatic hydrolysis of harvested biomass. The results showed that the strain PsJN stimulated plant growth in the earlier period of plant development and enlarged the cell size of stem piths, and it also indeed enhanced the essential metals uptake and accumulation in host plants. Moreover, we found that the PsJN-inoculated plant biomass released more glucose and xylose after hot water pretreatment and subsequent co-saccharification, which provided a novel insight into development of lignocellulosic biofuels from renewable biomass resources.

## Introduction

The global rise in energy consumption portends an increase in our future energy demands. It is thus urgent to develop efficient, sustainable and green energy production systems and biofuels is a leading technology in this field. Biofuels can be broadly categorized as first- and second-generation fuels that are derived from plant grains and oils, and lignocellulosic biomass, respectively (Chaturvedi and Verma, 2013). Recently, grain-based bioethanol production (a first-generation biofuel) is in question because it generates a dilemma over the use of agricultural crops and/or land for fuel vs. human food. Lignocellulosic biomass feedstocks (for the second-generation biofuel) are attractive because the human food concern is avoided and it is relatively low cost and sustainable (Agbor et al., 2011). To better meet the rapid increase in energy demands and more efficient utilization of restricted land, it is necessary to rapidly harvest more lignocellulosic biomass per unit time and/or area.

Lignocellulosic biomass is composed primarily of carbohydrate polymers (cellulose, hemicellulose) and the aromatic polymer (lignin) interwoven into the matrix of the cell wall (Ding et al., 2012). Polysaccharides are recalcitrant to depolymerization to simple sugars by enzymatic digestion, which can be fermented to liquid biofuels. Recalcitrance is also due, in part, to lignin protection of cellulose polymers from hydrolytic attack (Zeng et al., 2014). Natural biomass decay carried out by microbial communities effects the fragmentation of the cell wall lignin network to expose wall polysaccharides. Unfortunately, this process is too slow to meet the requirements of the large-scale biorefinery. Therefore, an efficient thermochemical pretreatment step is normally required prior to the enzymatic digestion of biomass and can be cost effective.

Meanwhile, plant engineering is an efficient approach to reduce the recalcitrance of biomass for thermochemical pretreatment and enzymatic digestion. Researchers at our institution, including some authors of this paper, have collaborated for some time with other groups in expressing heterologous cellulases in plants (Dai et al., 1999, 2005; Ziegler et al., 2000; Himmel et al., 2007; Sun et al., 2007; Taylor et al., 2008; Brunecky et al., 2011), and in conducting the chemical, physical and imaging characterization of native as well as genetic engineered plants (Penning et al., 2009; Ziebell et al., 2010; Brunecky et al., 2012; Bonawitz et al., 2014; Ciesielski et al., 2014a; Im Kim et al., 2014; Xiao et al., 2014). Most recently, inspired by the development of a pretreatment technology, which incorporates iron ions as co-catalysts in dilute acid and hot water pretreatments with enhanced release of simple sugars (Nguyen and Tucker, 2002; Liu et al., 2009; Wei et al., 2011; Degenstein et al., 2013; Kamireddy et al., 2013; Ciesielski et al., 2014b), our group used a genetic approach to overexpress the iron storage protein ferritin in plants (Wei et al., 2015). Remarkably, the obtained transgenic plants accumulated iron during plant growth, which not only effectively eliminated the time-consuming and costly step of loading iron ions into milled biomass prior to pretreatment, but also enhanced downstream biomass pretreatment and conversion to simple sugars (Wei et al., 2015).

To further implement the above approach for *in planta* iron accumulation, we speculate that the beneficial plant-microbial interaction can be utilized to simultaneously increase plant growth and biomass yield, and deliver metal catalysts for the downstream biomass pretreatment and conversion. Among the beneficial plant-microbial interactions, plant growth-promoting bacteria (PGPB) colonizing the rhizosphere or the internal tissues of some plant species benefit the host plants with the effects of including enhanced plant growth, improved tolerance to abiotic stresses, and reduced susceptibility to biological diseases (Poupin et al., 2013). PGPB have been applied in agriculture, horticulture, forestry and environmental restoration (Lucy et al., 2004), but few for the purpose of improving the quality of biomass feedstock used in biorefinery. Among the PGPB reported in literature, *Burkholderia phytofirmans* PsJN is a well-known strain that originally isolated from onion roots (Sessitsch et al., 2005). The following features of *B. phytofirmans* strain PsJN make it suitable for the studies to address the above purpose:

1. It establishes rhizospheric and endophytic colonization in various plants (Weilharter et al., 2011; Mitter et al., 2013; Zuniga et al., 2013). It has been proven to experimentally stimulate plant growth in seedlings or for short-term growth periods in a variety of plants such as grape, maize, potato, switchgrass, tomato, and wheat (Ait Barka et al., 2006; Da et al., 2012; Kim et al., 2012; Lowman et al., 2014; Naveed et al., 2014a,b; Wang et al., 2015).
2. Progresses have been made to understand the colonization process for this endophyte from the rhizosphere to above-ground parts of plants, which is important from the application as well as research perspectives. So far strain PsJN had been visualized efficiently colonizing the cortical cells, the endodermis and xylem vessels in primary roots of *Vitis vinifera*, and then moving to grape inflorescence stalks, pedicels and to young berries through xylem vessels (Compant et al., 2005, 2008). A recent report had demonstrated the colonization of PsJN in the roots and aerial organs of model plant *Arabidopsis* (Poupin et al., 2013).
3. For the model plant *Arabidopsis*, this strain can positively affect the whole life cycle of *A. thaliana* by increasing plant growth and accelerating growth rate in early ontogeny; as well as accelerating the flowering time and the appearance of senescence signs in later ontogeny, which shortens the vegetative period (Poupin et al., 2013). However, although most studies have assumed that phytohormone pathways, such as those induced by auxin and gibberellin, as well as by ethylene levels, were affected by PsJN producing ACC (1-aminocyclopropane-1-carboxylate) deaminase (Ahemad and Kibret, 2014), the actual mechanisms underpinning PsJN-plant interaction are poorly understood.
4. More specifically, recent genome analyses of *B. phytofirmans* PsJN in literature have identified the genes for the biosynthesis and excretion of siderophores (i.e., the molecules with high affinity for iron) as well as for the uptake of iron (Mitter et al., 2013); among these genes, the genetic and transcriptional analyses have been experimentally conducted for the ortholog genes, and the siderophore malleobactin have also been purified and characterized by mass spectrometry and bioassays in a close species *B. pseudomallei* (Alice et al., 2006). Most recently, the first transcriptome study of PsJN colonizing *in vitro* potato plants was published, which revealed that endophytic PsJN cells express a wide array of genes and pathways, including those point to iron acquisition and storage inside potato plants (Sheibani-Tezerji et al., 2015). The genomically predicted and transcriptionally demonstrated expression of these genes in *B. phytofirmans* PsJN prompted us to propose that PsJN-inoculation can improve the host plant's iron-uptake and accumulation, which can benefit the downstream plant biomass pretreatment and saccharification.

Thus, the objectives for this study are two-fold. *First*, it is to investigate the mechanism for *B. phytofirmans* PsJN-induced growth enhancement in the shoot tissues of *Arabidopsis* plants at physiological, morphological and anatomical levels. *Secondly*, it is also to specifically test the above genome and transcriptional analysis-driven proposal that PsJN-inoculation can improve the host plant's iron uptake and accumulation, which improves the downstream plant biomass pretreatment and conversion to simple sugars. To achieve these goals, we employed *B*. *phytofirmans* PsJN to inoculate *A. thaliana*, extracted RNA from the *Arabidopsis* shoots and for the second time in plants (after Sheibani-Tezerji et al., 2015) and the first time in *Arabidopsis*, demonstrated the expression of core *B. phytofirmans* genes related to iron storage and transport in PsJN-inoculated shoot tissues. We also measured the growth parameters and investigated the secondary tissue development of harvested stems. The results showed that the strain PsJN stimulated plant growth in the earlier period of plant development by enlarging the cell size of stem piths. In addition, we found that, also for the first time, PsJN-inoculated plant biomass had enhanced essential metals uptake with higher iron accumulation, and released more glucose and xylose after hot water pretreatment and subsequently co-saccharification. Here the data provide a novel insight into the mechanisms for the beneficial plant-microbial interaction that can be utilized for the development of cellulosic biofuels.

## Materials and methods

### Bacterial inoculum

*B*. *phytofirmans* PsJN, purchased from the German Collection of Microorganisms and Cell Culture (DSM #17436), was routinely grown in King's B liquid (KB) medium in 250-mL Erlenmeyer flasks, by incubating at 20°C, 150 rpm for 48 h, as described previously (Theocharis et al., 2012). Cell suspensions were collected by centrifugation to obtain the bacterial pellets. The bacterial pellets were washed twice with phosphate-buffered saline (PBS) (10 mM, pH 6.5), and resuspended in PBS again. Subsequently, the suspension was adjusted to approximate 10^4^ colony forming units (CFU)/mL and used as the inoculum (Poupin et al., 2013).

### Plant growth conditions and inoculation

*Arabidopsis thaliana* Col-0 seeds were surface sterilized with 70% ethanol for 1 min, followed by 1% commercial chlorine bleach and 0.01% Tween 20 solution for 7 min, and then washed three times with sterile distilled water. The sterilized seeds were then sown on 1% agar plates containing ½ Murashige and Skoog medium (MS) (Sigma-Aldrich) (Murashige and Skoog, 1962). Some plates were inoculated with bacteria. Eight seeds were sown in each plate and three plates were used for each treatment. Plates were vertically placed in the incubator at 24°C with a photoperiod of 12 h light and 12 h of dark. Several growth parameters were measured 10 days after sowing. For pot experiments, seeds were sterilized and immersed into bacterial suspension for 10 s, then sown into pots with sterile soils maintained at the same environmental conditions as described above for 2 months. Plants were watered with sterile water twice per week. Ten seeds were sown in each pot and three pots were used for each treatment. The same experiment was repeated three times.

### Bacteria re-isolation and PCR amplification

Roots of plantlets including inoculated and non-inoculated plants were removed from the agar plates or pots, and surface sterilized as described above. The sterilized root materials were placed in a sterile mortar and pestle containing 2 mL distilled water, and ground. Subsequently, the root tissue was removed and bacterial cells were collected in a 1.5-mL tube by differential centrifugation. Bacterial genomic DNA was extracted by using Fungal/Bacterial DNA MicroPrep™ Kits (Zymo Research Corporation, USA). These kits were used as templates to amplify gene *AcdS* with the primers *AcdS*-F (5′-TACAAAGCTTATGAACCTGCAACGATTCC-3′) and *AcdS*-R (5′- TATTCTAGATTGCCGTTGCGGAAAATG-3′) (Sun et al., 2009). The PCR products were checked using 0.8% agarose gel.

### RNA isolation from plant tissue

Stem tissues (50 mg) of 30-day plants with or without PsJN inoculation were harvested into 1.5 ml safe-Lock tubes (Cat. no. 022363204, Eppendorf North American, Hauppauge, NY) and immediately frozen into liquid nitrogen before RNA isolation. Frozen tissue was disrupted using a Bead mill (TissueLyser II, Cat. no. 85300, Qiagen, Valencia, CA, USA) at 30 Hz for 1 min using a single 5-mm stainless steel ball (Cat. no. 69989, Qiagen, Valencia, CA, USA). The homogenized sample was immediately subjected to RNA isolation, which was performed using RNeasy Plant Mini Kit (Cat. no. 74904, Qiagen, Valencia, CA, USA) and on-column DNA digestion with DNase I (Cat. no. 79254, Qiagen, Valencia, CA, USA) following the manufacturer's protocol. The yield and purity of isolated RNA was analyzed using NanoDrop ND-1000 spectrophotometer (Thermo Fisher Scientific, Grand Island, NY, USA) and the integrity was further checked by electrophoresis in a 1% agarose gel.

### Reverse transcription and cDNA synthesis

Purified and DNase-treated RNA samples from stem were subjected to plant rRNA depletion before reverse transcription. In brief, the plant rRNAs were depleted from the total RNA using a Ribo-Zero rRNA removal kit (Epicentre, Madison, WI, USA) as described by a most recent literature (Sheibani-Tezerji et al., 2015). The resultant bacterial RNA was reverse transcribed to cDNA using High-Capacity cDNA Reverse Transcription Kit (Cat. no. 4368814, Applied Biosystems, Grand Island, NY, USA) using random hexamers according to the manufacturer's instructions. The prepared cDNA samples were stored at −20°C until being used for the real-time RT (reverse transcription) PCR to analyze the transcriptional level of iron storage and transport related genes.

### Primer design and RT-PCR

Primers for *B. phytofirmans* 16S rRNA gene and genes related to iron storage and transport are listed in Table 1. These primers were designed by using the program ABI Primer Express version 3.0 (Applied. Biosystems, Foster City, CA) and specifying a T_m_ value between 58 and 62°C and an amplicon size between 100 and 250 bp. Power SYBR® Green PCR Master Mix (Cat. no. 4367659, Applied Biosystems, Grand Island, NY, USA) was used for RT-PCR analysis on 7300 Real-Time PCR System (Applied Biosystems, Grand Island, NY, USA). The RT-PCR consisted of an initial hold at 95°C for 10 min followed by 40 cycles of 95°C for 15 s and 60°C for 60 s. All reactions were performed in triplicate. The relative transcription level of genes was expressed as the “Ct (cycle threshold) value of interest gene—Ct value of *B. phytofirmans* 16S rRNA gene”; results are presented as the mean values of three biological repeats ± SEM (standard error of the mean).

**Table 1:** **Forward (F) and reverse (R) primer sequences for real time RT (reverse transcription)-PCR analysis of core genes of *B. phytofirmans* PsJN related to iron storage and transport**.

**New locus ID**	**Old locus ID**	**Gene name**	**Primer sequences and the sources**	**Amplicon size (bp)**
BPHYT_RS1733	Bphyt_R0054	16S rRNA	F: GATGCAACGCGAAAAACCTT R: CACCGGCAGTCTCCCTAGAG	200
BPHYT_RS01030	Bphyt_0219	Bacterioferritin	F: TGCGAGTCGGTAAGGGACTT R: ACCTTGCGGATCAGGTCGAT	110
BPHYT_RS06980	Bphyt_1412	Bacterioferritin	F: CTGCATGCGCGGATGTATAA R: TTCATCTCGCCGATCGATTC	80
BPHYT_RS16455	Bphyt_3313	Ferritin	F: CTGGCGGATCGAGGACATC R: CGGAGCCGCTTTCGATAAAC	110
BPHYT_RS03480	Bphyt_0714	Ferritin Dps family protein	F: AGCACGTCAATATCGGGATCA R: TGAGGTGCAGCGTGTTGAAC	150
BPHYT_RS28420	Bphyt_5727	Ferritin Dps family protein	F: CGCTTTATCGCGAACTGCTT R: TCGCCTGCGTGCATTTCTT	100
BPHYT_RS20295	Bphyt_4079	TonB-dependent siderophore receptor	F: GGTGCTGGCGTACAGATTGC R: CAACACGGGCACCATCAAG	130
BPHYT_RS22965	Bphyt_4626	TonB-dependent siderophore receptor	F: GCTTCGCTGCCGTTGAAATT R: GATGTGATCTACGGGCCGTTT	170
BPHYT_RS20270	Bphyt_4074	L-ornithine 5-monooxygenase (PvdA)	F: AAAAGCCGCTTCACGTTCAT R: TGACGGTTTGCCCGTAGTTC	160

*All the primers were designed in this study*.

### Content analysis for metals in biomass

Sixty-day-old *A. thaliana* Col-0 seedlings were collected and air-dried at room temperature. The shoots without seeds were then ground to pass through a 20-mesh (1 mm) screen using a Wiley Mill (Thomas-Wiley, Philadelphia), and used to measure the concentrations of metal ions by the Chemical Analysis Laboratory at the University of Georgia, using the methods of nitric acid digestion and inductively coupled plasma-optical emission spectroscopy (ICP-OES) (Wheal et al., 2011).

### Microscopy and image analysis

For sample preparation, 60-day-old fresh *A*. *thaliana* seedlings cultivated in the soil pots were cropped at the stem base. Four segments were generated by cutting the cropped *A*. *thaliana* seedlings at different positions of 4, 12, 20, and 28 cm from the stem base, respectively. Transverse sections of each segment were sectioned into about 50 μm-slices by hand-cutting using a single-blade razor. Cell size of the prepared samples was determined by using the optical microscope. For microscopy and image analysis, an inverted microscope with digital camera (Olympus IX71 with DP70 digital camera, Melville, NY USA) was used. All images were recorded at a resolution of 4080 × 3072 pixels and analyzed using the ImageJ software.

### Biomass pretreatment and conversion to sugars

The samples were ground as described above. Five micrograms ground samples were weighed into an individual Hastelloy wells on a 96-well plate, followed by hot water pretreatment at 180°C for 17.5 min, and subsequently enzymatic saccharification at 40°C for 70 h using Novozymes Cellic® CTec2 (3 mg enzyme/g biomass), Following enzymatic saccharification, the glucose released was measured using an glucose oxidase/peroxidase (GOD-POD) assay. Xylose release was measured using a xylose dehydrogenase (XDH) assay (Selig et al., 2010; Gao et al., 2013).

### Statistical analysis

The obtained data throughout this study were statistically analyzed by Student's *t*-test using Microsoft Excel 2013, including for the calculation of the means and standard errors.

## Results and discussion

### Data mining for *B. phytofirmans* core target genes related to iron storage and transport

As described in the Introduction section, a most recent transcriptome analysis of *B. phytofirmans* PsJN colonizing *in vitro* potato plants revealed that endophytic PsJN cells express a wide array of genes and pathways (Sheibani-Tezerji et al., 2015). In total, out of the 7311 putative genes annotated in the genome, 4591 transcripts of *B. phytofirmans* PsJN were detected in PsJN-colonized potato shoot tissues, indicating 63% of the genome-predicted genes were actively expressed. These active genes include five ferritin-encoding genes and two TonB-dependent siderophore receptor encoding genes; these genes, along with their RPKM (reads per kilobase per million mapped) values detected in the PsJN-colonized potato shoot tissues under normal growth condition, were retrieved from literature (Sheibani-Tezerji et al., 2015), and were listed in Table 2 of this paper.

**Table 2:** **Expression level in *B. phytofirmans* PsJN-inoculated shoot tissues for the core *B. phytofirmans* genes related to iron storage and transport**.

**New locus ID**	**Old locus ID**	**Gene name**	**Sheibani-Tezerji et al., 2015**	**This study**
			RPKM in PsJN-inoculated potato shoots	Ct in PsJN-inoculated Arabidopsis shoots
**GENES FOR FERRITINS**				
BPHYT_RS01030	Bphyt_0219	Bacterioferritin	48.4	–
BPHYT_RS06980	Bphyt_1412	Bacterioferritin	290.3	–
BPHYT_RS16455	Bphyt_3313	Ferritin	863.6	7.5 ± 1.3
BPHYT_RS03480	Bphyt_0714	Ferritin Dps family protein	258.6	24.4 ± 2.3
BPHYT_RS28420	Bphyt_5727	Ferritin Dps family protein	294.0	9.5 ± 1.2
**GENES FOR SIDEROPHORE BIOSYNTHESIS AND RECEPTORS**				
BPHYT_RS20295	Bphyt_4079	TonB-dependent siderophore receptor	16.4	–
BPHYT_RS22965	Bphyt_4626	TonB-dependent siderophore receptor	0.58	11.2 ± 1.1
BPHYT_RS20270	Bphyt_4074	L-ornithine 5-monooxygenase (PvdA)		10.0 ± 0.7

*As a comparison, the RPKM (reads per kilobase per million mapped) values of expressed genes in the non-stressed samples of PsJN-inoculated potato shoots based on literature were also presented (Sheibani-Tezerji et al., 2015). For this study, the relative expression level of genes in PsJN-inoculated Arabidopsis shoots was expressed as the “Ct (cycle threshold) value of interest gene—Ct value of B. phytofirmans 16S rRNA gene”; data are presented as the mean values of three biological repeats ± SEM*.

In addition to the above set of *B. phytofirmans* genes expressed in PsJN-colonized potato shoot tissues, special efforts were also made in this study to identify the gene encoding L-ornithine 5-monooxygenase (EC 1.14.13.195), which catalyzes the conversion of L-ornithine to *N*^5^-hydroxyornithine (see reaction equation below), the first step in the biosynthesis of all hydroxamate-containing siderophores, such as pyoverdin.

$$\begin{array}{l} \text{L-ornithine}+\text{ NADPH}+{\text{O}}_{2}\overset{\text{L-ornithine 5-monooxygenase}}{\to }N^{5}\text{ -}\\ {\text{ hydroxy-L-ornithine+NADP}}^{+}+{\text{H}}_{2}\text{O} \end{array}$$

Previous literature had demonstrated that the L-ornithine 5-monooxygenase gene in *B. pseudomallei* (BPSL1776) is involved in the biosynthesis of siderophore (Alice et al., 2006). Using BPSL1776 as query, BlastP search against *B. phytofirmans* genomes revealed that the *B. phytofirmans* gene Bphyt_4074 has 73% similarity with that of BPSL1776, thus *B. phytofirmans* gene Bphyt_4074 is also added to the list of core *B. phytofirmans* genes related to iron acquisition, transport and storage in Table 2. Such gene homolog search is confirmed by a similar BlastP analysis of using *B. phytofirmans* Bphyt_4074 as a query in Uniprot database (www.uniprot.org), which showed that Bphyt_4074 had a similarity of 50–80% with that of L-ornithine 5-monooxygenase PvdA in *B. cepacia, B. glathei, Pseudomonas fluorescens, P. putida*, and *P. syringae* etc. Among them, the function of above *pvdA* genes in siderophore production had been experimentally demonstrated in *B. cepacia* (Sokol et al., 1999), *B. pseudomallei* (Alice et al., 2006) and *P. aeruginosa* (Visca et al., 1994; Putignani et al., 2004; Ge and Seah, 2006; Meneely and Lamb, 2007). Together, these eight genes listed in Table 2 were tested for their expression in PsJN-colonized Arabidopsis shoot tissues in this study, as described in following two sections.

### Expression of *B. phytofirmans* target genes in *Arabidopsis* shoots

The ferritins are a family of protein cages that play a key role in iron storage, and are ubiquitously found in animals, plants, and microorganisms. The ferritins in bacteria can be categorized into three sub-families (Zhang and Orner, 2011): (1) the classical 24-mer ferritins that do not contain heme moieties, and can store up to ~4500 Fe atoms (Theil, 1987); (2) the heme-containing bacterioferritins; and (3) the DNA-binding proteins from starved cells (Dps) that are 12-mer (thus also called mini-ferritin), and can store up to ~500 Fe atoms (Grant et al., 1998). *B. phytofirmans* possess all these three types of ferritin genes in their genome, which includes at least one classic ferritin (Bphyt_3313), two bacterioferritin (Bphyt_0219, Bphyt_1412), and two mini-ferritin Dps (Bphyt_0714, Bphyt_5727) genes (Table 2). Out of them, the classic ferritin (Bphyt_3313) and two mini-ferritin Dps (Bphyt_0714, Bphyt_5727) genes of *B. phytofirmans* were detected in the PsJN-inoculated *Arabidopsis* shoot, with a relative Ct values between 7.5 and 24.4 (Table 2); for which the Ct value for 16S ribosomal RNA (reference gene) is set to be zero.

Meanwhile, the TonB-dependent siderophore receptor (Bphyt_4626) and L-ornithine 5-monooxygenase (PvdA; Bphyt_4074) genes of *B. phytofirmans* were also detected in the PsJN-inoculated *Arabidopsis* shoot, with a relative Ct values being 11.2 and 10.0, respectively (Table 2).

### Comparison of *B. phytofirmans* target gene expressions in PsJN-inoculated *Arabidopsis* vs. potato shoots

As described in the above sections, a most recent publication examined the transcriptome of *B. phytofirmans* PsJN colonizing *in vitro* potato plants (Sheibani-Tezerji et al., 2015); the specific genes related to iron acquisition and storage function are listed in Table 2. Note that due to the fact that different research groups used different plants' shoot samples, it is challenging to conduct a vis-a-vis comparison between the gene expression of this study and that in literature. Nevertheless, since these two studies used a similar procedure for RNA and cDNA preparations (including a common depletion step to remove plant host rRNA), a comparison is still meaningful to reveal the commonly or differentially expressed genes related to iron acquisition and storage function.

As illustrated in Table 2, three ferritin genes (Bphyt_3313, Bphyt_0714 and Bphyt_5727) and one TonB-dependent siderophore receptor gene (Bphyt_4626) were detected in both studies, suggesting they are likely to be the “house-keeping” ferritins of this endophyte. Interestingly, the classic ferritin gene (Bphyt_3313) was found to be the most abundant ferritin gene in both potato and *Arabidopsis* shoots (Table 2); this ferritin gene is worthy of further study.

Interestingly, L-ornithine 5-monooxygenase (PvdA; Bphyt_4074) genes of *B. phytofirmans* was detected in *Arabidopsis* shoots (Table 2), but not in the potato shoots by previous study (Sheibani-Tezerji et al., 2015), warrantying future studies.

### Implication for *B. phytofirmans* target gene expression

Previous reports have demonstrated the colonization of PsJN in the roots and aerial organs of model plant *Arabidopsis* (Poupin et al., 2013). The current study further demonstrated the expression of 16S rRNA gene and the genes related to the iron storage and transport of this strain in *Arabidopsis* shoot. The colonization of PsJN cells along the path from rhizosphere and roots to shoots, will likely facilitate the transport of iron from its source (i.e., the rhizosphere) to the sink (i.e., plant shoots), thus supports our proposal that PsJN enhances iron accumulation in host plants.

It is noteworthy that the effects of PsJN inoculation on the transcriptome of host *Arabidopsis* had been examined by a previous study, which found that the expression of 408 *Arabidopsis* genes was altered (Poupin et al., 2013). However, the expression of ferritin genes and other genes related to iron transport were not changed. While such observation should not affect the above proposed contribution of endophytes to iron accumulation in host plants, it may indirectly limit the extent of the proposed PsJN-inoculation enhancement on iron accumulation.

### *B. phytofirmans* PsJN stimulated the growth of *A. thaliana*

**Figure 1:**
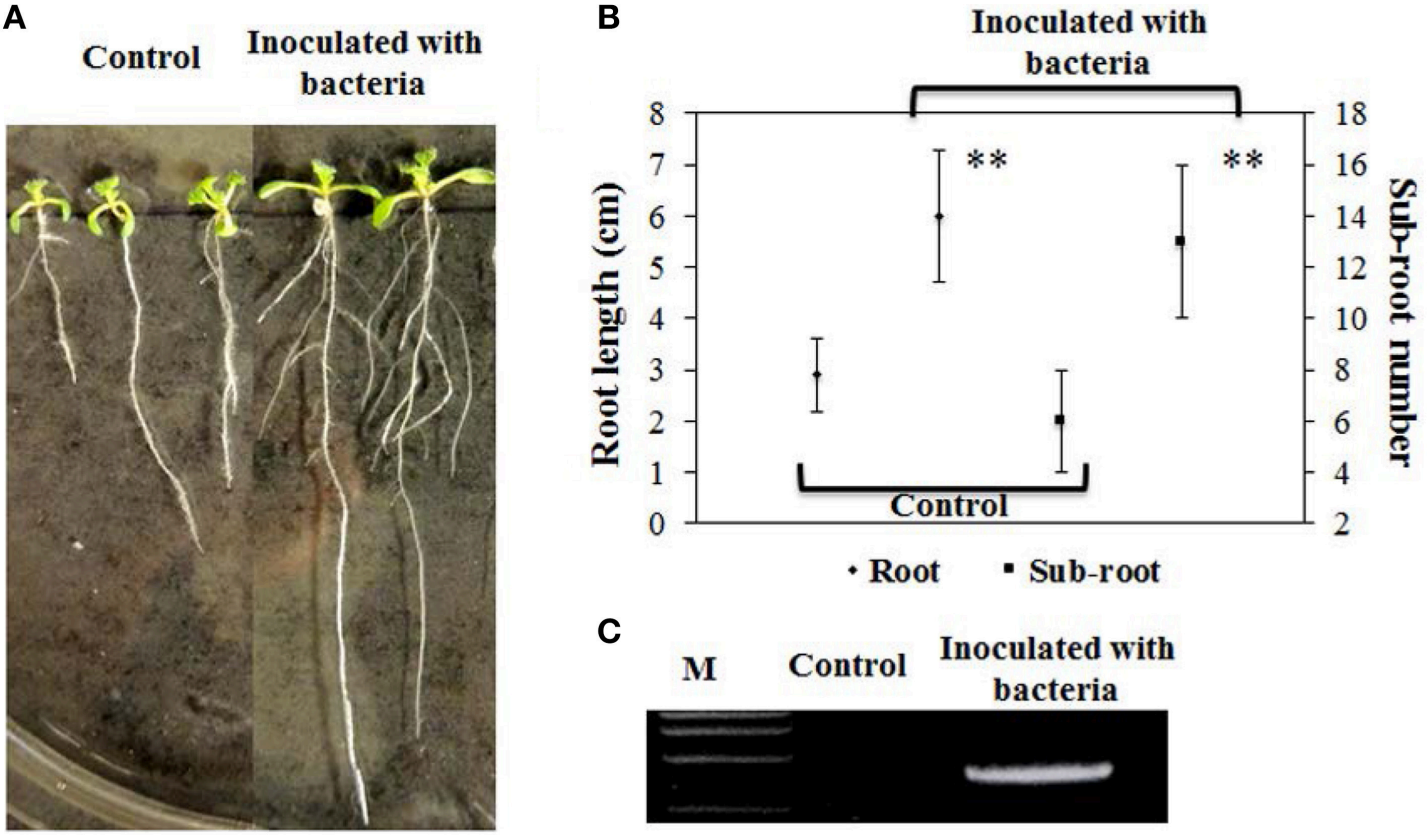
**Effects of *B*. *phytofirmans* on root growth of representative *A*. *thaliana* at 10 days after inoculation and PCR analysis**. **(A,B)** Root elongation and number of sub-root of PsJN-inoculated and non-inoculated (control) plants. Values were presented as the mean ± SD of seven plants for each line. ^**^indicated that significant difference from non-inoculated (control) plants at *P* < 0.01 by Student's *t*-test. **(C)** PCR analysis of endophytic PsJN colonization in plants.

We employed *B*. *phytofirmans* PsJN to inoculate *A*. *thaliana* and investigated plant morphology throughout the whole life cycle. Ten days after inoculation of ½MS medium agar plates with *B*. *phytofirmans* PsJN, primary root length of *A*. *thaliana* inoculated with PsJN was significantly elongated (~110%), compared to non-inoculated control, or 6.0 cm and 2.9 cm, respectively (*P* < 0.01). These results are consistent with those reported previously (Zuniga et al., 2013). Surprisingly, the data obtained in this study showed that the number of sub-roots of inoculated plants was over two times higher than that of the control, or 13 vs. 6, respectively (*P* < 0.01; Figures 1A,B). It was previously reported that some rhizobacteria, including *Burkholderia* strains, produced ACC deaminase, mediated ethane levels in plants, and may enable the increased number of lateral roots (Shahzad et al., 2010). Also, the plant hormones gibberellins and IAA, induced by *B. phytofirmans* PsJN, had the ability to promote lateral root formation and/or extension (Poupin et al., 2013; Vacheron et al., 2013; Zuniga et al., 2013). Consequently, it was presumed that these results were caused by the synergistic interaction of phytohormones and secondary metabolites secreted by rhizobacteria and the host plant.

In addition, strain PsJN was re-isolated from the roots of inoculated *A*. *thaliana* inoculated with PsJN and its genomic DNA was then extracted. A PCR for the amplifying probe gene *acdS*, using the extracted genomic DNA as a template, was successfully performed to produce a clear 1035-bp band in inoculated plant roots, whereas no band was found in control, demonstrating the successful rhizospheric and endophytic colonization of plants (Figure 1C).

**Figure 2:**
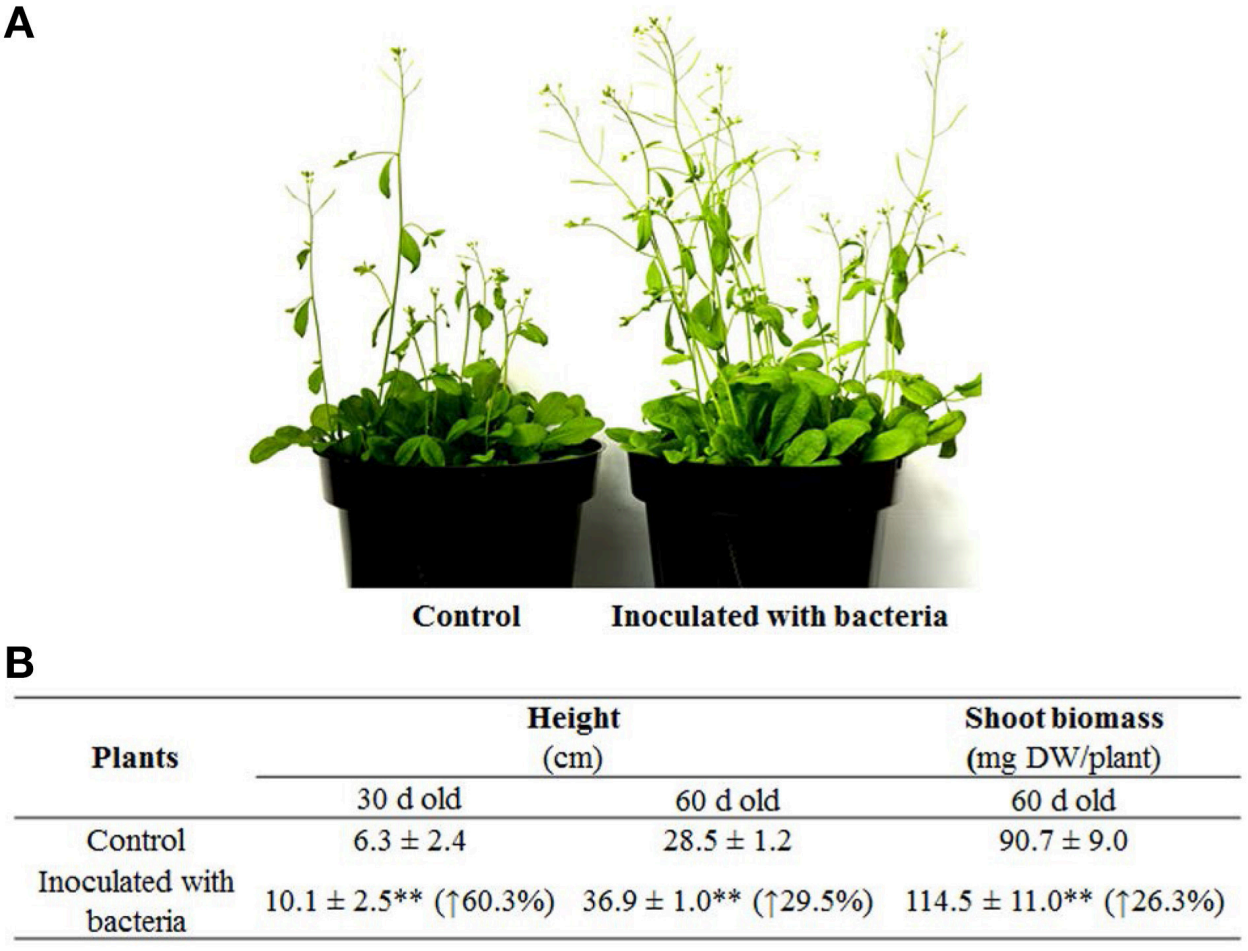
**Growth of representative plants inoculated with *B*. *phytofirmans* PsJN and the non-inoculated (control) plants. (A)** Pictures for 30-day-old *A*. *thaliana*, **(B)** Data for plant height and shoot biomass weight. Values were presented as the mean ± SD of at least 10 plants for each line. The percentage values inside brackets were the increase in inoculated lines compared with the control; ^**^indicated that significant difference from non-inoculated (control) plants at *P* < 0.01 by Student's *t*-test.

Subsequently, long-term effects of strain PsJN on *A*. *thaliana* grown in pots were also investigated by assessing the standard growth parameters. Plants inoculated with PsJN exhibited a significant increase in plant height compared to control (*P* < 0.01): at 30 days after inoculation, i.e., the mean plant height promoted by PsJN was 10.1 cm compared to 6.3 cm for the control. Furthermore, after 60 days of inoculation, the mean plant heights of inoculated and non-inoculated plants were recorded as 36.9 and 28.5 cm, respectively (Figures 2A,B). The dry weight for shoots of the 60-day-old plants was then weighed and the results showed that dry weight per plant of inoculated plants was about 25% heavier than that of control (i.e., 114.5 and 90.7 mg (*P* < 0.01), respectively). Similar results have been obtained using other plants, including switchgrass, grapevine, and potato inoculated with PsJN in various growth environments with approximate 0.6-, 6- and 2-fold increase of biomass yields over controls (Frommel et al., 1991; Ait Barka et al., 2006; Kim et al., 2012).

Interestingly, the increase of PsJN-inoculated plant height compared to non-inoculated control declined from 60.3% at 30 days to 29.5% at 60 days after inoculation, suggesting that strain PsJN significantly accelerated growth rate during the first half of plant development and then the growth rate level declined, meeting the same level as the non-inoculated controls in the examples of long-term interaction (Poupin et al., 2013). Furthermore, PsJN also accelerated the flowering and senescence time (Poupin et al., 2013). Such results suggest that strain PsJN could shorten the plant vegetative period and demonstrate e greater biomass yields at a given growth time as a consequence, possibly, of better acquisition of nutrients and/or enhanced plant metabolism by the endophytic bacterium (Ait Barka et al., 2006; Poupin et al., 2013).

### *B. phytofirmans* PsJN modified anatomical organization of stems

**Figure 3:**
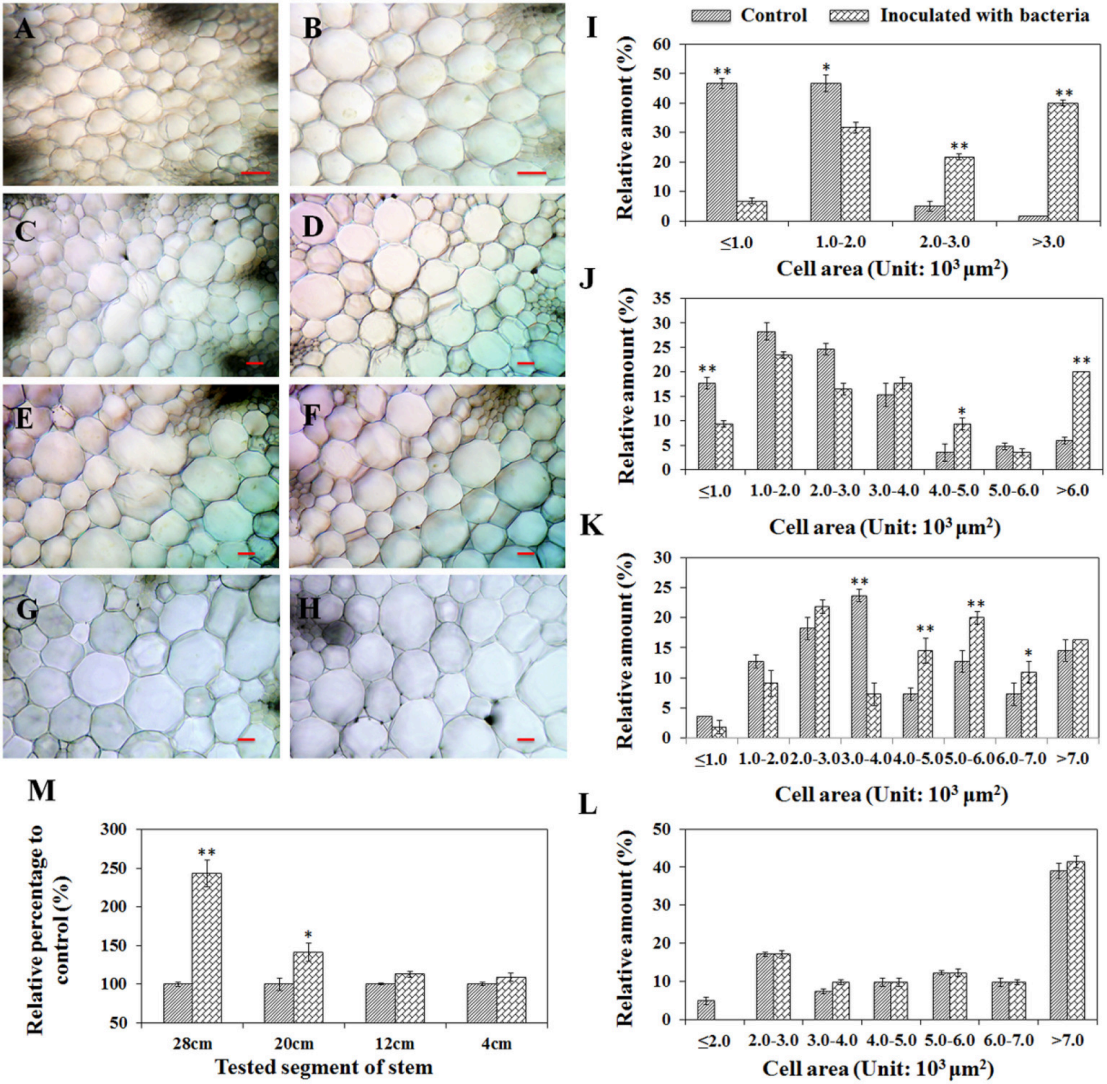
**Cell size comparisons of the pith of *A*. *thaliana* inoculated with *B*. *phytofirmans* PsJN and non-inoculated control plants**. Shoots of 60-day-old plants were transversely cut from 28 cm **(A,B)**; 20 cm **(C,D)**; 12 cm **(E,F)** and 4 cm **(G,H)** over the base, respectively. Among them, **(A,C, E,G)** represented variation of the cell sizes of the pith from PsJN inoculated plants, and the others represented control plants. Line = 100 μm. In **(I–M)**, where the cell area was measured, the same number of cells from samples inoculated with bacteria and controls was randomly chosen in the center of the piths in each treatment. Data were the mean ± SD from three replications, each with at least 40 cells. ^*^ and ^**^ respectively indicated that significant difference from non-inoculated (control) plants at *P* < 0.05 and *P* < 0.01 by Student's *t*-test.

To understand the effects of plant stem cell modification caused by PsJN, a comparison of the mean pith cell size from inter-fascicular cells between treatments was performed by studying the cross sections of stems taken at four defined distances (28, 20, 12, and 4 cm) from the stem base. A significant increase of pith cell size for PsJN-inoculated *A*. *thaliana* compared to non-inoculated control tissue was observed at all sections, except for the 4 cm sample (Figures 3A–H). To quantify and catalog pith cell size distribution, for each treatment, the same number of cells (at least 40) located at the pith center were randomly chosen and used to measure cell area. Cell area measurement revealed that a shift to larger cell size populations for PsJN inoculated-stem samples (except for the 4 cm samples) compared to control treatments. Cell areas of 4000 to 6000 μm^2^, over 4000 μm^2^, and over 2000 μm^2^ significantly increased in the cross sections of 12, 20, and 28 cm (Figures 3I–L, *P* < 0.01). Furthermore, the total tested cell area was also investigated and the results showed that PsJN inoculated samples taken at 28 cm and 20 cm were significantly larger than non-inoculated controls, with the relative percentage to control being 243% and 141% (*P* < 0.01), respectively. The other samples were consistent with the results of controls (Figure 3M).

It is noteworthy that the double wall thickness in the inter-fascicular cells cannot be determined precisely in the prepared cross sections of stem samples due to the limitation of optical microscope. In literature, transmission electron microscope (TEM) coupled with image processing software has been used to measure the cell wall thickness (Zhu et al., 2015). Future studies on this aspect may provide additional information for assessing the effects of PsJN inoculation on plant cell anatomy.

According to these results, *B*. *phytofirmans* PsJN probably stimulated plant growth by enlarging the cell size in the pith. The plant height data obtained from different growing phases indicates that the plant growth enhancement occurred primarily in the earlier period of plant development. In literature, related observations of changing of rosette areas in PsJN-inoculated plants during growth drew a similar conclusion (Poupin et al., 2013). Although the actual mechanism of PGPB-plant symbiosis is not yet clear, it is generally believed that the PGPB facilitate plant growth directly by assisting in resource acquisition (nitrogen, phosphorus, and essential minerals) or by modulating plant hormone levels, or indirectly by decreasing the inhibitory effects of various pathogens.

### *B. phytofirmans* PsJN enhanced metal content uptake in *A. thaliana*

**Figure 4:**
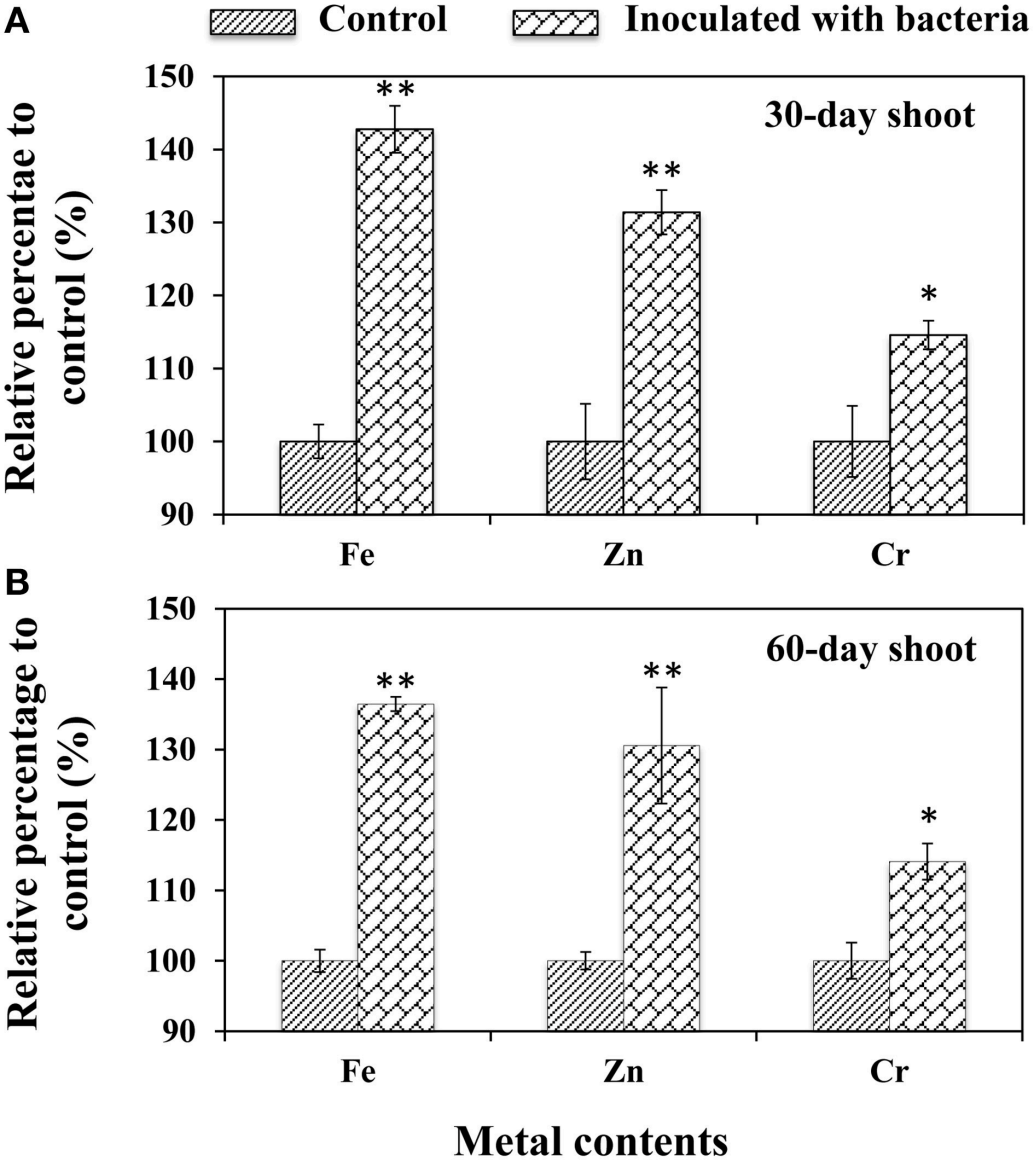
**Accumulation of metal content in the shoots of *A*. *thaliana***. Shoots were harvested from 30-day-old **(A)** and 60-day-old **(B)** plants, respectively. Values are presented as the mean ± SD of three replicates. The tested samples came from at least ten plants for each line. ^*^ and ^**^ respectively indicated that significant difference from non-inoculated (control) plants at *P* < 0.05 and *P* < 0.01 by Student's *t*-test.

To validate the effects of *B*. *phytofirmans* on the metal ion uptake ability of plants, the titers of 20 metal ions, including Al, As, B, Ba, Ca, Cd, Co, Cr, Cu, Fe, K, Mg, Mn, Na, Ni, P, Pb, Si, Sr, and Zn, were determined in the shoots of 30- and 60-day-old *A*. *thaliana*. The results showed that only three trace metals: Fe, Zn, and Cr were significantly increased compared to CK control (Figure 4), suggesting that PGPB promoted plant growth by enhancing the uptake of certain micronutrients. Among them, Fe was the most easily available by PsJN-inoculated plants (i.e., 42.8 and 36.5% higher than that in control for 30- and 60-day-old shoots, respectively; *P* < 0.01), followed by Zn (31.4% for 30.6% for 30- and 60-day-old shoots, respectively; *P* < 0.01) and Cr (14. 6% and 14. 1% for 30- and 60-d-old shoots, respectively; *P* < 0.05) (Figure 4).

It has been suggested that the increase of iron content may depend on the siderophores that are low molecular mass iron chelators with high association constants for complex iron as an optimal mechanism for bacteria to absorb iron (Ahemad and Kibret, 2014) are secreted by *B*. *phytofirmans* PsJN (Sun et al., 2009). Plants are also known to assimilate iron from bacterial siderophores by means of different mechanisms, such as ligand exchange reactions and chelation/release of iron (Ahemad and Kibret, 2014). Beyond iron, siderophores also form stable complexes with the other heavy metals, such as Zn and Cr, to help plants absorb beneficial micronutrients or tolerate the toxicity of heavy metals (Ahemad and Kibret, 2014). However, in the current study, all treated plants were cultivated in normal soils used in gardening without the addition of a metal fertilizer.

Furthermore, the results above suggest that strain *B*. *phytofirmans* PsJN can potentially be applied in phytoremediation of heavy metal-contaminated soils. In fact, more recently, PGPB composed primarily of genera *Pseudomonads* and *Acinetobacter* have been successfully applied to reduce plant stress in metal-contaminated soils by enhancing nutrient absorption and improving plant detoxification of metals (Tak et al., 2013). Historically, however, reports of the applications of *B*. *phytofirmans* to phytoremediation have been scarce, although a recent report showed that *B*. *phytofirmans* PsJN stimulated faster tree growth by removing contaminants, such as high amounts of Fe and Cr from soil irrigated with textile effluent (Afzal et al., 2014).

### *B. phytofirmans* PsJN accelerated pretreatment and enzymatic hydrolysis of biomass

**Figure 5:**
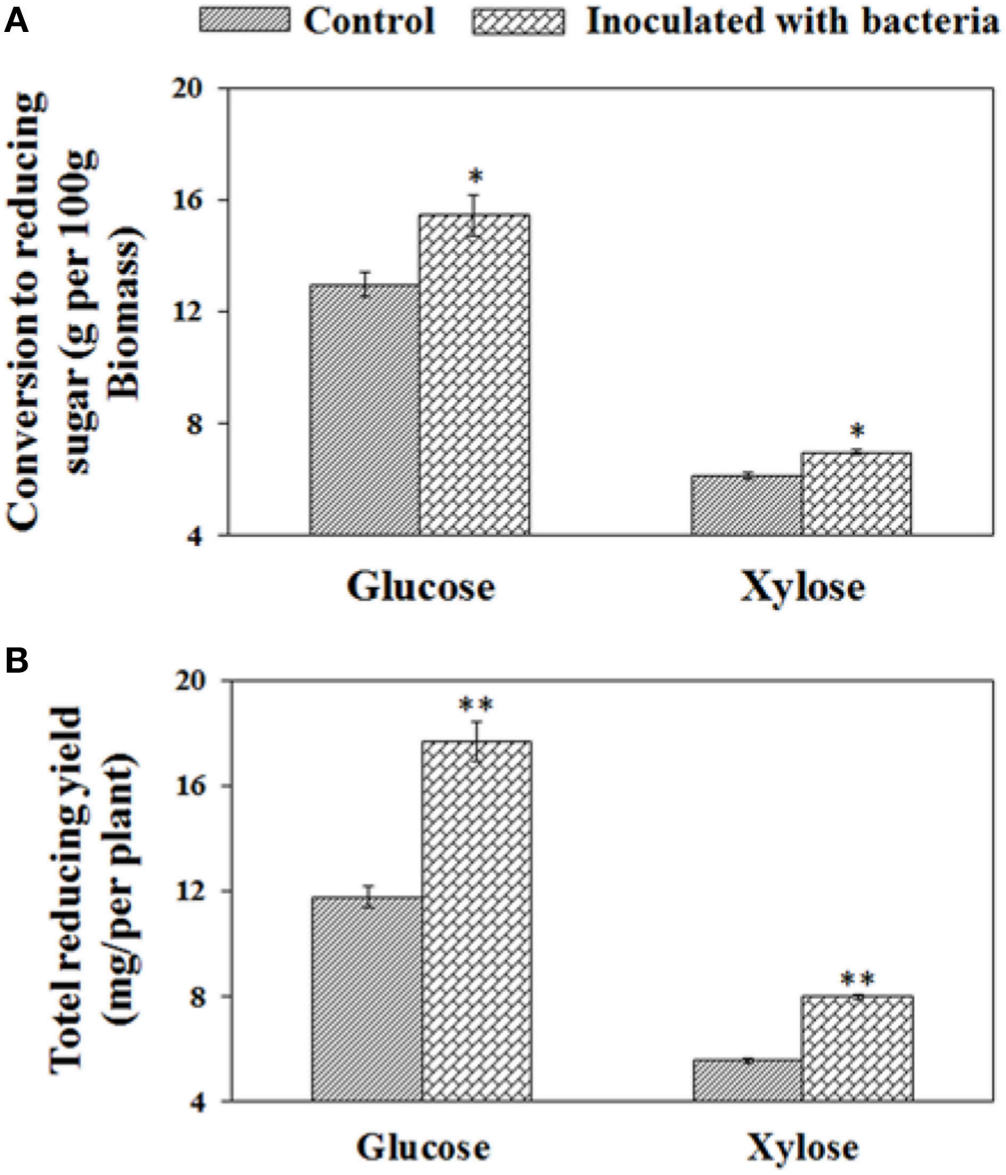
**Conversion to reducing sugar from biomass after pretreatment and subsequent saccharification for 70 h**. The conversion yields of reducing sugars are presented on the basis of biomass dry weight **(A)** and per plant **(B)**, respectively. Values are presented as the mean ± SD of three replicates for each pretreatment, and the harvested biomass came from at least ten plants for each line. ^*^ and ^**^ respectively indicated that significant difference from non-inoculated (control) plants at *P* < 0.05 and *P* < 0.01 by Student's *t*-test.

To investigate the effects of *B*. *phytofirmans* PsJN on enzymatic hydrolysis of biomass from both treatments, the harvested biomass was initially pretreated by liquid hot water at 180°C for 17.5 min and subsequently enzymatically saccharified for 70 h with the addition of Novozyme Cellic® CTec2. Compared with control plant biomass, the PsJN-inoculated plant biomass were more easily converted to xylose and glucose after pretreatment and the subsequent enzymatic saccharification, showing enhancements of 14.8 and 19.4%, respectively (i.e., 15.4 g and 12.9 g glucose per 100 g biomass) and (7.0 g and 6.1 g xylose per 100 g biomass), respectively. By considering biomass yield at a target harvesting time, overall each PsJN-inoculated plant could convert 51.3% and 44.4% more glucose and xylose (i.e., 17.7 mg and 11.7 mg glucose, and 7.97 mg and 5.52 mg xylose) (see Figure 5), respectively. Consequently, PGPB *B*. *phytofirmans* PsJN had a positive effect on the enzymatic digestion rate and release of reducing sugar.

The relative cell wall polymer compositions, including cellulose, hemicellulose and lignin may have been changed in PsJN-inoculated plants as shown by a shift to larger pith cells size, whereas no significant change of cell wall thickness of the inter-fascicular cells during plant growth, leading to the high content of polysaccharides in pith cell walls. The pith cell wall, also called the parenchyma-type second wall (pSW), was partially lignified and contained more polysaccharides (Ding et al., 2012; Handakumbura and Hazen, 2012). Therefore, after hot water pretreatment, enzymatic digestion rates of PsJN inoculated plant biomass were higher than the control, and eventually released more reducing sugar at the same saccharification time. In addition to cell wall compositions, more iron uptake in PsJN-inoculated plants possibly also played an enhancement role in biomass conversion. Ion-assisted pretreatment is known to enable an increase in solubilization and enzymatic digestibility of polysaccharides by affecting multiple components of the cell wall, including the C-O-C and C-H bonds in cellulose, leading to the enhancement of xylan removal and lignin relocation (Wei et al., 2011). However, the actual chemical mechanisms defining this process should be further investigated by multiple technologies.

## Conclusion

The present study revealed the expression of genes related to iron storage, siderophore biosynthesis and transport in the shoot tissues of PGPB strain *B*. *phytofirmans* PsJN-inoculated *Arabidopsis*. We measured a range of important parameters to investigate the effects of PsJN on plant growth and subsequent biomass conversion to sugars. The increase of plant height and biomass yield resulting from PsJN-inoculation demonstrated that *B*. *phytofirmans* PsJN had a positive effect on plant growth. Plant cell anatomy analysis also showed that larger plants contained enlarged stem pith cells in the earlier stage of plant development. Moreover, determination of plant mineral nutrients suggested that PsJN-inoculated plants might stimulate growth by absorbing essential mineral nutrients, such as Fe, Zn, and Cr. Additionally, biomass hot-water pretreatment and subsequent enzymatic saccharification indicated that *B*. *phytofirmans* PsJN enhanced Arabidopsis plant biomass deconstruction to release more reducing sugars, including glucose and xylose. These findings enhance our understanding of the effects of the PGPB strain on plant growth and biomass deconstruction, and provide a foundation for further studies to investigate the cytological and molecular mechanisms of *Arabidopsis*-endophyte interaction. Future studies on relatively abundant PsJN ferritin gene (Bphyt_3313) and siderophore biosynthesis gene (Bphyt_4074) may further shed light on their roles in enhancing iron accumulation in host plants.

## Author contributions

SD and HW designed and coordinated the study and revised the manuscript. SZ conducted the plant growing and parameters determination, microcopy and imaging analysis, and prepared the manuscript draft. HW conducted mineral nutrients determination, pretreatment and subsequent co-saccharification. SD, HW, MT, and MH contributed to experimental design, data analysis and manuscript revision. All authors have read and approved the final manuscript.

### Conflict of interest statement

The authors declare that the research was conducted in the absence of any commercial or financial relationships that could be construed as a potential conflict of interest.
